# DNA-PK_cs_ promotes chromatin decondensation to facilitate initiation of the DNA damage response

**DOI:** 10.1093/nar/gkz694

**Published:** 2019-08-09

**Authors:** Huiming Lu, Janapriya Saha, Pauline J Beckmann, Eric A Hendrickson, Anthony J Davis

**Affiliations:** 1 Division of Molecular Radiation Biology, Department of Radiation Oncology, UT Southwestern Medical Center, Dallas, TX 75390, USA; 2 Department of Biochemistry, Molecular Biology, and Biophysics, University of Minnesota Medical School, Minneapolis, MN 55455, USA

## Abstract

The DNA damage response (DDR) encompasses the cellular response to DNA double-stranded breaks (DSBs), and includes recognition of the DSB, recruitment of numerous factors to the DNA damage site, initiation of signaling cascades, chromatin remodeling, cell-cycle checkpoint activation, and repair of the DSB. Key drivers of the DDR are multiple members of the phosphatidylinositol 3-kinase-related kinase family, including ataxia telangiectasia mutated (ATM), ataxia telangiectasia and Rad3-related (ATR), and the DNA-dependent protein kinase catalytic subunit (DNA-PK_cs_). ATM and ATR modulate multiple portions of the DDR, but DNA-PK_cs_ is believed to primarily function in the DSB repair pathway, non-homologous end joining. Utilizing a human cell line in which the kinase domain of DNA-PK_cs_ is inactivated, we show here that DNA-PK_cs_ kinase activity is required for the cellular response to DSBs immediately after their induction. Specifically, DNA-PK_cs_ kinase activity initiates phosphorylation of the chromatin factors H2AX and KAP1 following ionizing radiation exposure and drives local chromatin decondensation near the DSB site. Furthermore, loss of DNA-PK_cs_ kinase activity results in a marked decrease in the recruitment of numerous members of the DDR machinery to DSBs. Collectively, these results provide clear evidence that DNA-PK_cs_ activity is pivotal for the initiation of the DDR.

## INTRODUCTION

DNA double-stranded breaks (DSBs) are deleterious DNA lesions that if left unrepaired or are misrepaired can lead to mutations and chromosomal aberrations linked to carcinogenesis (1). To cope with DNA damage including DSBs, cells have evolved complex mechanisms collectively termed the DNA damage response (DDR) (2). The DDR for DSBs includes recognition of the damaged DNA, initiation of cellular signaling cascades, recruitment of DNA repair proteins to the damage site, remodeling of the chromatin near the DSB, activation of cell-cycle checkpoints, and repair of the DSB (3). Ultimately, the DDR drives multiple cellular decisions, including the choice of the appropriate pathway to repair the DSB, the decision between apoptosis or senescence if unresolved DSBs persist, modulation of transcription, and activation of heightened immune surveillance (4). The importance of the DDR is unequivocal and is underscored by the fact that defects in the DDR can result in predisposition to cancer, premature aging, and other diseases, like disorders in the nervous, immune, and reproductive systems (2–4).

Three members of the phosphatidylinositol-3-kinase-like kinase (PIKK) family, DNA-dependent protein kinase catalytic subunit (DNA-PK_cs_), ataxia telangiectasia-mutated (ATM), and ataxia telangiectasia-mutated and Rad3-related (ATR), are instrumental in driving the DDR in response to DSBs (5). DNA-PK_cs_ and ATM are activated by DSBs, whereas ATR responds to a broad spectrum of DNA damage that is processed to generate single-strand DNA (ssDNA), such as DSBs that are induced by damage interfering with DNA replication. All three kinases are recruited to the site of the DNA damage by DNA damage sensors, which promotes activation of their catalytic activity (6). DNA-PK_cs_ is recruited to DSBs by the Ku heterodimer, which consists of the Ku70 and Ku80 subunits, and the interaction between Ku70/80 and DNA-PK_cs_ requires the presence of double-strand DNA (7). The complex formed at the DSB consisting of DNA, Ku70/80, and DNA-PK_cs_ is referred to as the DNA–PK complex or simply, DNA–PK. Recruitment of ATM to chromatin in response to DSBs is mediated by the Meiotic Recombination 11–Radiation Sensitive 50–Nijmegen Breakage Syndrome 1 (MRE11–RAD50–NBS1; MRN) complex. ATR is recruited to ssDNA through its binding partner, ATR Interacting Protein (ATRIP), which indirectly recognizes ssDNA through an interaction with the ssDNA-binding protein replication protein A (RPA).

The main function of ATM and ATR is to drive signal transduction pathways in response to DNA damage (5). ATM and ATR show functional redundancy and their functions are likely intertwined. ATM is rapidly activated by DSBs and phosphorylates a significant number of factors to stimulate numerous sections of the DDR (8). Subsequently, there is an ATM > ATR switch. This is driven by the resection of the DSB end and RPA loading onto the ssDNA generated by this process that results in ATR activation, allowing it to maintain phosphorylation of some of ATM’s substrates (9). Phospho-proteomic studies have identified several hundred proteins that are phosphorylated in response to DSBs induced by ionizing radiation (IR), with the phosphorylation of almost all these proteins attributed to the activity of ATM and ATR (10–12). DNA-PK_cs_ is rapidly recruited to DSBs and is activated, but the direct functionality of DNA-PK_cs_ in the DDR appears to be limited to its role in regulating DSB repair via the non-homologous end joining (NHEJ) pathway (7). Since DNA-PK_cs_ is rapidly activated by IR-induced DSBs and has similar substrate specificities as ATM and ATR *in vitro*, it seems likely that DNA-PK_cs_ may be responsible for phosphorylating a number of the proteins identified in the above-mentioned phospho-proteomic screens. Although there is a long list of proteins that are excellent targets of DNA-PK_cs_*in vitro*, a clear understanding of the functional relevance of many of these phosphorylation events is annoyingly lacking (7,13). This is further complicated by the fact that the DNA damage responsive PIKKs can compensate for the loss of each other (5). For example, DNA-PK_cs_ phosphorylates several ATM target proteins in ATM-defective cells, revealing functional redundancy between DNA-PK_cs_ and ATM (14,15).

To determine a potential role for DNA-PK_cs_ catalytic activity in DDR signaling in response to DSBs, we characterized a human cell line, HCT116, in which a point mutation was introduced to generate a kinase-dead version of the DNA-PK_cs_ protein. Since DNA-PK_cs_ is recruited to DSBs faster than ATM and ATR (16–18), we focused on identifying functions of DNA-PK_cs_ immediately after induction of IR-induced DSBs. Here, we report that immediately after DSB induction DNA-PK_cs_ is responsible for phosphorylating the histone protein, H2AX and the chromatin remodeling factor, KAP1. Moreover, we found that DNA-PK_cs_ catalytic activity is required for chromatin remodeling at early time points post-irradiation and promotes the rapid recruitment of DDR proteins to DSB sites. Combined, our findings reveal that DNA-PK_cs_ enzymatic activity plays a direct role in the initiation of the DDR.

## MATERIALS AND METHODS

### Cell lines and cell culture

HCT116, IBR3, AT5BIVA, and U2OS cell lines were cultured in Hyclone α-minimum Eagle's medium supplemented with 5% newborn calf serum, 5% fetal bovine serum (Sigma) and 1× penicillin/streptomycin (Gibco) and grown in an atmosphere of 5% CO_2_ at 37°C. To inhibit DNA-PK_cs_ or ATM, cells were incubated for 2 h prior to irradiation with 10 μM NU7441 (SelleckChem) or 10 μM KU60019 (SelleckChem), respectively.

### Construction of a DNA-PK_cs_ kinase-dead mutant cell line

Wild-type (WT) HCT116 cells were sequentially targeted with two rAAV gene targeting vectors, one containing flanking LoxP sites to create a conditional WT exon 79 of DNA-PK_cs_, and a second to create a point mutation in exon 79. This point mutation results in a lysine to arginine substitution at amino acid 3753, which deleteriously affects γ-phosphate transfer and ATP positioning in the DNA-PK_cs_ kinase domain (16,19,20). Vector arms were amplified from WT HCT116 DNA and fused to a neomycin resistance construct with a high fidelity polymerase (Invitrogen) using primers (described in Supplementary Table S1) and cloned into a pAAV-MCS vector. Complete viral particles were produced in AAV-293 cells as described (21).

**Figure 1.**
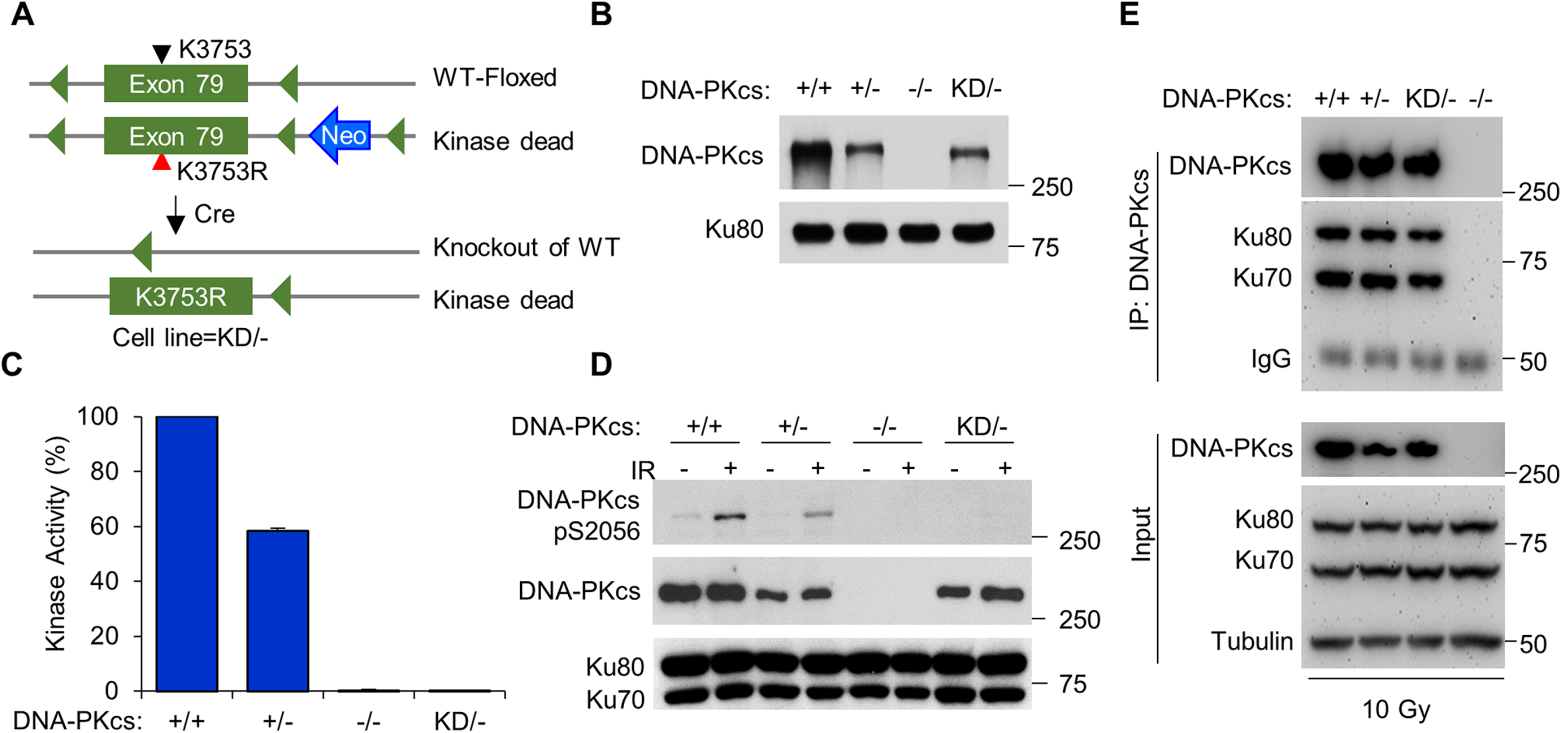
Generation and initial characterization of the DNA-PK_cs_ kinase-dead (KD) HCT116 cell line. (**A**) Basic schematic of the method used to generate the HCT116 DNA-PK_cs_ kinase dead (KD/−) cell line. (**B**) Expression level of DNA-PK_cs_ in HCT116 DNA-PK_cs_ +/+, +/−, −/−, and KD/− cells as assessed by western blotting. (**C**) Measurement of DNA-PK_cs_*in vitro* kinase activity. Nuclear extracts from the HCT116 DNA-PK_cs_ +/+, +/−, −/−, and KD/− cells were examined for their ability to phosphorylate a biotin-tagged H2AX peptide. H2AX phosphorylation was observed in the −/− cell line and this was subtracted from the other samples’ readouts. The 100% kinase activity was normalized using the +/+ cell lysate results. The data are presented as the mean ± SD from three individual experiments. (**D**) Measurement of DNA-PK_cs_*in vivo* kinase activity. The HCT116 DNA-PK_cs_ +/+, +/−, −/−, and KD/− cell lines were mock-treated or γ-irradiated with a dose of 10 Gy and allowed to recover for 30 min. Cell extracts were prepared and western blot analysis was performed to assess autophosphorylation of DNA-PK_cs_ at serine 2056. Immunoblotting of Ku70 and Ku80 were used as loading controls. (**E**) The interaction between DNA-PK_cs_ and the Ku70/80 heterodimer is not affected by inactivating the kinase activity of DNA-PK_cs_. DNA-PK_cs_ was immunoprecipitated from the HCT116 +/+, +/−, −/−, and KD/− cell lines 5 min after being irradiated with 10 Gy of γ-rays. The immunoprecipitates were analyzed by western blotting with anti-DNA-PK_cs_, Ku80, and Ku70 antibodies. Tubulin was used as a loading control for the input of each immunoprecipitation assay.

WT HCT116 cells were transduced with the conditional exon 79 rAAV vector and single cell colonies were isolated using G418 resistance as a selection criteria. A clone containing a conditional exon 79 in DNA-PK_cs_ was then treated with Cre recombinase to remove the neomycin resistance cassette. A clone lacking the neomycin resistance cassette but still containing the conditional exon 79 allele was subsequently subjected to a second round of targeting using the point mutation vector and repeated selection in G418. Single cell subcloning of G418-resistant colonies resulted in cell lines that were harvested and analyzed by polymerase chain reaction (PCR) for correct incorporation of the K3753R mutation within exon 79 of DNA-PK_cs_. These cells were then treated with Cre recombinase a second time to (i) remove the conditional allele of DNA-PK_cs_ and (ii) the neomycin resistance gene, generating a cell line that contained only allele of DNA-PKcs and that allele contained the kinase-dead mutation (Figure 1A). Absence of a WT allele was verified by PCR and DNA sequencing using primers described in Supplementary Table S1.

### Cell irradiation and immunoblotting

Cells were irradiated with γ-rays generated by a Mark 1 ^137^Cs irradiator (JL Shepherd and Associates) with a total dose of 10 Gy unless otherwise indicated. Following mock treatment or irradiation, the cells were allowed to recover for the time indicated in the figure legends, washed three times with ice-cold phosphate-buffered saline (PBS), and then whole-cell extracts were generated by resuspending the cells in 1× radioimmunoprecipitation assay (RIPA) buffer (Cell Signaling Technology), sonicated, and then incubated on ice for 20 min. After centrifugation at 14 000 rpm at 4°C for 30 min to clear the lysate, the protein concentration of each sample was measured using the Pierce BCA Protein Assay kit (Thermo Fisher). Each sample (30 μg) was separated via sodium dodecyl sulfate-polyacrylamide gel electrophoresis (SDS-PAGE) and then transferred to a polyvinylidene difluoride (PVDF) membrane. The PVDF membrane was blocked using 5% non-fat milk and the membranes were then incubated with primary antibodies for 2 h at room temperature. The membranes were washed four times with tris-buffered saline-Tween (TBS-T), then incubated with secondary antibodies at room temperature for 2 h. Following the washing of the membranes four times with TBS-T, the immunoblots were reacted using Pierce™ enhanced chemiluminescent (ECL) Western Blotting Substrate (Thermo Fisher), exposed to Blue X-ray films (Light Labs), and then developed using a Protec X-ray film processor. The films were scanned and the band intensity were quantified with Image J version 1.51 where indicated. All quantified data are derived from at least three independent experiments.

### Antibodies

The following commercial antibodies were used: anti-DNA-PK_cs_ phospho-S2056 (Abcam, ab124918), anti-MRE11 (Abcam, ab214), anti-RAD50 (BD Bioscience, 611010), anti-NBS1 (BD Bioscience, 611871), anti-ATM (Abcam, ab109027), anti-ATM phospho-S1981 (Abcam, ab81292), anti-MDC1 (Abcam, ab5003), anti-KAP1 (Bethyl Laboratories, A300–274A), anti-KAP1 phospho-S824 (Bethyl Laboratories, A300–767A), anti-phospho-H2AX (S139) (EMD Millipore, 05–636), anti-γH2AX (Cell Signaling Technology, 7631), anti-tubulin (Sigma, T5168), anti-CHK2 phospho-T68 (Cell Signaling Technology, 2197), anti-CHK2 antibody (Cell Signaling Technology, 2662), anti-XLF (Cell Signaling Technology, 2854), anti-LIG4 antibody (Cell Signaling Technology, 14649), anti-Histone H3 antibody (Cell Signaling Technology, 9715), anti-XRCC4 antibody (Santa Cruz, sc-271087), and anti-EXO1 (Thermo Fisher Scientific, MA5–12262). In-house produced antibodies include mouse monoclonal antibodies against DNA-PK_cs_ (25–4), Ku80, and Ku70. Secondary antibodies included anti-mouse IgG (HRP-linked) and anti-rabbit IgG (HRP-linked), which were purchased from Cell Signaling Technology.

### Cell proliferation assay

HCT116 DNA-PK_cs_ +/+, +/−, −/−, or KD/− cells were seeded (3 × 10^3^) in each well of a 6-well tissue culture dish. The increase in cell number was determined by counting via Beckman Coulter Z2 Coulter^®^ Particle Count and Size Analyzer at daily intervals starting at Day 4 and ending on Day 7 post-plating. The results are presented as Mean ± SD, from three biological repeats with a *P*-value that was obtained using a Student *t*-test.

### *In vitro* DNA-PK_cs_ kinase assay

Each kinase reaction contained five times kinase buffer (125 mM Tris–HCl, pH 7.9, 125 mM MgCl_2_, 5 mM DTT, 125 mM KCl, and 50% glycerol), 100 ng of sonicated herring DNA, 20 nM Ku70/80, 0.16 μM [γ-^32^P] ATP (6000 Ci/mmol), 1 μg biotin-labeled H2AX (biotin-AVGKKASQASQEY) and 10 μg of nuclear extract from HCT116 DNA-PK_cs_ +/+, +/−, −/−, or KD/− cell lines. The final volume was brought to 10 μl. Reactions were incubated for 30 min at 30°C and terminated by the addition of 1 μl 0.5 M ethylenediaminetetraacetic acid (EDTA). The biotinylated-H2AX peptide was captured using a SAM2 Biotin Capture Membrane (Promega) and the membrane was washed following the manufacturer's suggested protocol. H2AX phosphorylation was detected by PhosphoImager analysis (Amersham Biosciences) and scintillation counting. Background phosphorylation of H2AX was observed in the −/− cell line and this was subtracted from the other samples’ readouts. 100% kinase activity was normalized using the +/+ cell lysate results. The reported results are derived from three independent experiments.

### Co-immunoprecipitation

For the immunoprecipitation experiments, HCT116 DNA-PK_cs_ +/+, +/−, −/−, and KD/− cell lines were irradiated with 10 Gy and allowed to recover for 5 min. The cells were washed with cold PBS, harvested, and then lysed using immunoprecipitation (IP) Lysis buffer (50 mM Tris–HCl pH 7.4, 250 mM NaCl, 2 mM MgCl_2_, 0.4% NP-40, 0.6% Triton X-100, 1× protease inhibitor cocktail, 1× phosphatase inhibitor cocktail 2, 20 U/ml Benzonase (Novagen)) on ice for 20 min. The lysates were sonicated and then cleared by centrifugation at 14 000 rpm at 4°C for 30 min and protein concentrations were obtained using the Pierce BCA Protein Assay kit (Thermo Fisher). A total of 2 μg protein were incubated with 2 μg DNA-PK_cs_ antibody and 30 μl of Protein A/G magnetic agarose beads (Thermo Fisher) overnight with spinning at 4°C. The beads were washed with IP washing buffer (20 mM Tris–HCl pH 7.4, 200 mM NaCl, 0.2% Triton X-100) five times, and boiled in one time SDS sample buffer. The immunocomplexes were resolved via SDS-PAGE and western blot analysis was performed as described above using the antibodies specified in the figure legends.

### Colony formation assay

Cell survival curves were obtained by measuring the colony-forming abilities of irradiated cell populations (22). Cells were irradiated at doses of 1, 2, 4, or 6 Gy and then plated on 60-mm plastic Petri dishes. After 10 days, cells were fixed with 100% ethanol and stained with 0.1% crystal violet in a 100% ethanol solution. Colonies were scored and the mean value for triplicate culture dishes was determined. Cell survival was normalized to plating efficiency of untreated controls for each cell type.

### Neutral comet assay

For the neutral comet assay, HCT116 DNA-PK_cs_ +/+, +/−, −/−, and KD/− cells were either mock treated or irradiated with 10 Gy of IR, and the cells were then allowed to recover for 30 min and subsequently placed on ice. The neutral comet assay was performed according to the manufacturer's protocol (CometAssay kit, R&D systems). OpenComet software v1.3.1, an ImageJ plug-in, was used for automated analysis of the comets. At least 400 comets for each condition were analyzed for plotting the comet tail moment data and statistical analyses.

### Fluorescent immunostaining and microscopy

All fluorescent immunostaining and microscopy experiments were performed as previously described (22,23). For immunostaining of IR-induced MDC1 foci, cells were grown on coverslips 1 day before the experiment. The cells were mock-treated or irradiated with 2 Gy of IR and then allowed to recover for 5 or 10 min. The cells were subsequently washed twice with cold 1× PBS and fixed with 4% paraformaldehyde (in 1× PBS) for 20 min at room temperature. The cells were washed four to five times with 1× PBS and incubated in 0.5% Triton X-100 on ice for 10 min. Cells were washed four to five times with 1× PBS and incubated in blocking solution (5% goat serum (Jackson Immuno Research) in 1× PBS) overnight. The blocking solution was then replaced with the MDC1 (Abcam, ab5003) primary antibody diluted in 5% goat serum in 1× PBS and incubated for 2 h at room temperature. Cells were then washed five times with immunofluoresence (IF) wash buffer (1% BSA in 1× PBS) and then incubated with the Alexa Fluor 488 (Molecular Probes) secondary antibody in 1% BSA and 2.5% goat serum in 1× PBS for 1 h in the dark. Following five washes with IF wash buffer, the cover slip was mounted in VectaShield mounting medium containing 4′,6-diamidino-2-phenylindole (DAPI). The images were acquired using a Zeiss Axio Imager fluorescence microscope utilizing a 63× oil objective lens. The foci were analyzed and counted using Imari (Bitplane) image analysis software.

### NHEJ and HR reporter assay

The plasmids for GFP-based reporter assays, pimEJ5GFP and pDRGFP, were obtained from Addgene in order to investigate NHEJ and HR efficiency, respectively (24,25). 1 × 10^6^ HCT116 DNA-PK_cs_ +/+, +/−, −/−, or KD/− cells were transiently transfected with 5 μg pCMV-ISceI, 200 ng DsRed (Clontech) and either 2 μg pimEJ5GFP or pDRGFP via Lonza Solution V using program D-032. Cells were analyzed using a BD FACSCalibur™ using the parameters previously established (24,25). The results are presented as Mean ± SEM, from three biological repeats with *P*-values obtained by a Student *t*-test.

### Subcellular fractionation

The association of DDR proteins to chromatin following IR-induced DNA damage was examined using the Thermo Fisher Subcellular Protein Fractionation Kit as described (26). Briefly, the HCT116 DNA-PK_cs_ +/−, −/−, and KD/− cells were mock-treated or irradiated with 10 Gy and allowed to recover for 10 min. Next, the cells were harvested after trypsinization and processed with the Thermo Fisher Subcellular Protein Fractionation Kit according to the manufacturer's instructions. The protein concentration of each sample was measured using a Pierce BCA Protein Assay kit (Thermo Fisher) and 30 μg protein of each sample was separated via SDS-PAGE, and then transferred to a PVDF membrane for western blotting analysis using the protocol outlined above.

### Micronuclease (MNase) digestion assay

Nucleosome relaxation assays were performed as described with some modifications (27). Briefly, in 10 cm dishes, HCT116 DNA-PK_cs_ +/−, −/−, and KD/− or AT5 cells were grown to 90% confluency and were either mock-treated or incubated for 2 h prior to irradiation with 10 μM NU7441 (SelleckChem) or 10 μM KU60019 (SelleckChem) to inhibit DNA-PK_cs_ or ATM, respectively. Next, the cells were mock-treated or irradiated with a γ-ray dose of 10 Gy and allowed to recover for 10 min. Following the incubation, the cells were washed with ice-cold PBS and then resuspended in ice-cold hypotonic Hank's balanced salt solution (HBSS) buffer (340 mM sucrose, 15 mM Tris, pH 7.5, 15 mM NaCl, 60 mM KCl, 10 mM DTT, 0.15 mM spermine, 0.5 mM spermidine and 0.5% Triton X-100) and allowed to incubate on ice for 10 min with periodic vortexing. The nuclei were pelleted via centrifugation at 11 000 rpm for 5 min at 4°C, washed with HBSS buffer, resuspended in 1:1 HBSS/glycerol, and stored at −20°C overnight. Subsequently, the nuclei were centrifuged and resuspended in 75 μl MNase digestion buffer (250 mM sucrose, 15 mM Tris, pH 7.5, 15 mM NaCl, 60 mM KCl, 0.5 mM DTT and 1 mM CaCl_2_). The MNase digestion was performed at 25°C after adding 1.7 μl 1 U/ml MNase to 75 μl of resuspended nuclei for 3 min and stopped by adding 1.5 μl of 0.5 M EDTA and 8 μl of 5% SDS with 1 mg/ml Proteinase K (Sigma-Aldrich), and each sample was incubated at 37°C for 30 min. The DNA was extracted with phenol/chloroform/isoamyl-alcohol and precipitated with isopropanol and sodium acetate. After measuring the DNA concentration, 2 μg of each sample was separated on a 1.2% agarose gel electrophoresed in Tris-acetate EDTA buffer, stained with ethidium bromide, and visualized via the Bio-Rad ChemiDoc™ MP Imaging System. The DNA band intensity of each sample were quantified with Image J version 1.51 and normalized to total volume of the whole lane.

### Laser micro-irradiation and live cell imaging

GFP-NBS1, GFP-EXO1, GFP-Ku70, GFP-XLF, or GFP-XRCC4 were transfected into HCT116 DNA-PK_cs_ +/+, −/−, or KD/− cells with JetPrime^®^ (Polyplus) following the manufacturer's instructions. Twenty-four hours after the transfection laser micro-irradiation and real-time recruitment was performed with a Carl Zeiss Axiovert 200M microscope with a Plan-Apochromat 63X/NA 1.40 oil immersion objective (Carl Zeiss) as previously described (28). DSBs were generated with a 365-nm pulsed nitrogen laser (Spectra-Physics), which was directly coupled to the epifluorescence path of the microscope (28). Time-lapse images were taken via a Carl Zeiss AxioCam HRm camera. The cells were maintained in a CO_2_-independent medium (Invitrogen) at 37°C during micro-irradiation and time-lapse imaging. Fluorescence intensities of the micro-irradiated area and control area were determined by Carl Zeiss Axiovision software, v4.5, and the intensity of irradiated was normalized to non-irradiated control area as previously described (26).

## RESULTS

### Generation of a human cell line that harbors a point mutation that inactivates DNA-PK_cs_ kinase activity

The kinase activity of DNA-PK_cs_ is required for the cellular response to DSBs, but its exact role in this process, outside from modulating NHEJ, is largely unknown. To identify novel functions for DNA-PK_cs_ activity in human cells, we utilized a rAAV-mediated gene targeting strategy to introduce a point mutation in the DNA-PK_cs_ gene (*PRKDC*) that inactivates the kinase domain of DNA-PK_cs_ in the human colon carcinoma cell line HCT116 (Figure 1A). Specifically, lysine 3753, which is involved in the γ-phosphate transfer and ATP positioning in the active site of DNA-PK_cs_, was mutated to arginine (K3753R) to inactive DNA-PK_cs_ catalytic activity (16,19,20). The gene targeting strategy resulted in one allele of DNA-PK_cs_ with the K3753R mutation and a knock-out of the second allele, resulting in a ‘kinase dead’ (KD) cell line with the genotype of DNA-PK_cs_^KD/−^ (see ‘Materials and Methods’ section for a full description of the gene targeting strategy). Comparison of HCT116 cells with wild-type DNA-PKcs^+/+^ (+/+), heterozygous DNA-PKcs^+/−^ (+/−), null DNA-PKcs^−/−^ (−/−) (29), or kinase dead (KD/−) showed that the KD/− cell line has approximately the same level of DNA-PK_cs_ protein as the +/− cell line, and about half the amount of the +/+ cell line, consistent with the notion that the KD/− cell line does not express protein from one allele (Figure 1B). The −/− cell line showed a marked growth delay compared to the +/+ and +/− cell lines, which is in support of previous data (Supplementary Figure S1A) (29). The KD/− cell line grew slightly slower than the +/+ and +/−, but this difference was not statistically significant (Supplementary Figure S1A).

The loss of DNA-PK_cs_ kinase activity was verified using an *in vitro* DNA-PK kinase assay using cell lysates derived from DNA-PK_cs_ +/+, +/−, −/−, and KD/− cell lines (Figure 1C and Supplementary Figure S1B). Furthermore, IR-induced autophosphorylation of DNA-PK_cs_ at serine 2056 (DNA-PK_cs_ pS2056) was completely lost in the KD/− cell line, which verified that the DNA-PK_cs_ protein generated in the KD/− cell line is in fact functionally inactive (Figure 1D). At last, co-immunoprecipitation assays showed that the KD protein still interacted with the Ku70/80 complex after irradiation in a fashion similar to the wild-type protein (Figure 1E), which confirms the belief that the loss of DNA-PK_cs_ activity in the KD/− cell line is due to the point mutation in DNA-PK_cs_ and not from a loss of the interaction between DNA-PK_cs_ and the Ku heterodimer.

### The kinase activity of DNA-PK_cs_ is required for cell survival and DSB repair after IR

**Figure 2.**
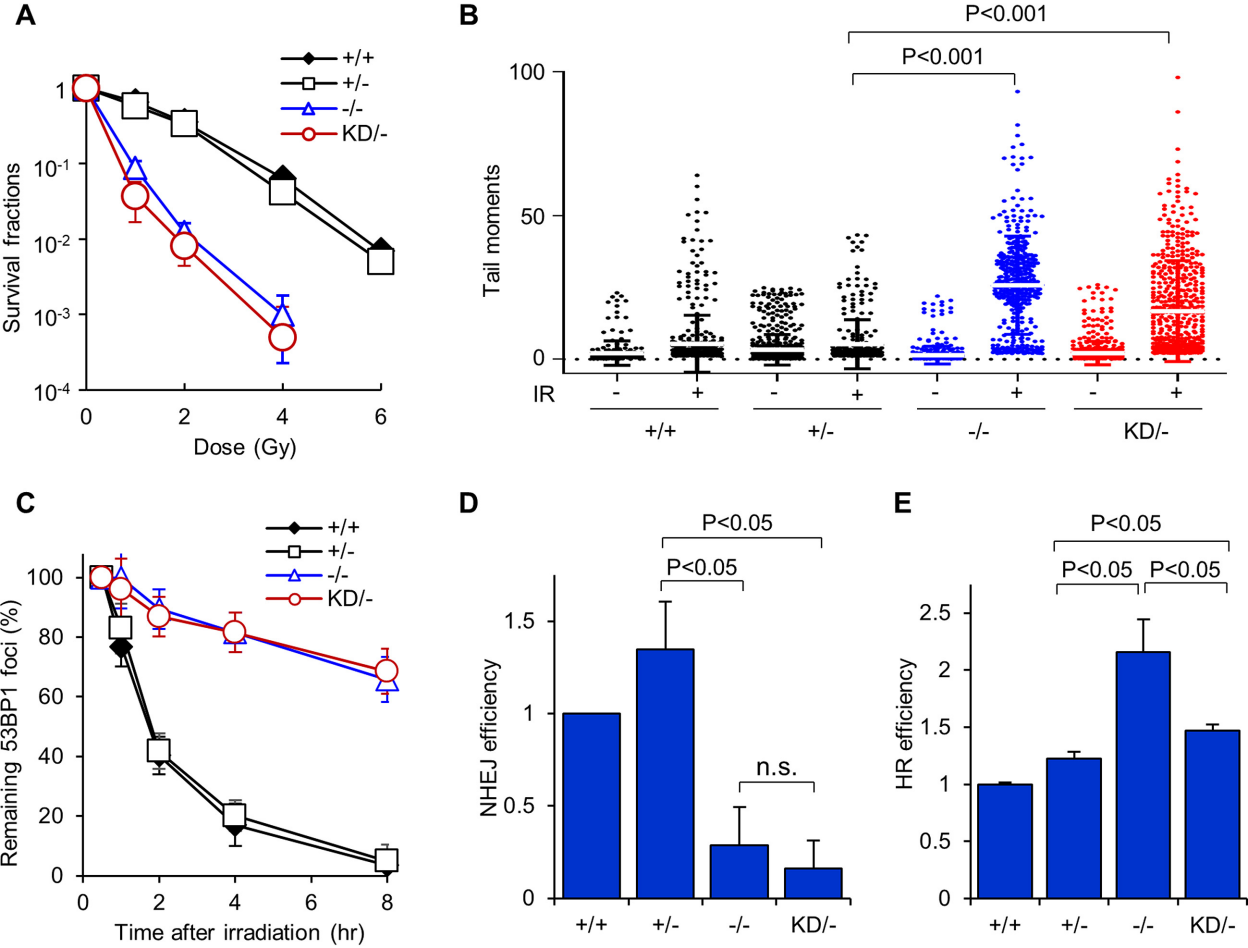
DNA-PK_cs_ kinase activity is important for cell survival and DSB repair following DSB induction. (**A**) Colony formation assays were performed to compare radiation sensitivities of the HCT116 DNA-PK_cs_ +/+, +/−, −/−, and KD/− cell lines. Cells lines were left cycling and irradiated at the indicated doses and plated for analysis of survival and colony-forming ability. The data is presented as mean ± SD from three independent experiments. (**B**) DSB repair proficiency of HCT116 DNA-PK_cs_ +/+, +/−, −/−, and KD/− was evaluated using neutral Comet assay. Cells were irradiated with 10 Gy of IR, allowed to recover for 30 min, harvested, and then Comet assays were performed. Tail moment values for >400 mock treated and irradiated cells were calculated and plotted via a distribution dot plot. (**C**) Dynamic 53BP foci distribution in HCT116 DNA-PK_cs_ +/+, +/−, −/−, and KD/− cells after IR. The cells were irradiated with 1 Gy of γ-rays and 53BP1 foci formation and resolution was assessed 0.5, 1, 2, 4 and 8 h later. Data were normalized to the foci number enumerated at 30 min post IR. Remaining foci number per time point were calculated and plotted. Error bars denote SEM of three independent experiments. (**D**) NHEJ-mediated and (**E**) HR-mediated DSB repair were evaluated in HCT116 DNA-PK_cs_ +/+, +/−, −/−, and KD/− cells using GFP-based reporter assays. The data were presented as Mean ± SEM with *P*-values from at least three biological repeats.

In rodents, the inactivation of DNA-PK_cs_ kinase activity results in increased radiosensitivity and decreased DSB repair capacity (19,20,30). To test whether KD/− human cells were also impaired in their ability to repair DSBs, we first assessed the cellular response to radiation. Clonogenic survival assays in asynchronous populations revealed that the KD/− and −/− cell lines were markedly more radiosensitive than the +/+ and +/− cells (Figure 2A). The KD/− cell line was consistently slightly more radiosensitive than the −/− cell line, but this difference was not statistically significant. To confirm that the KD/− cell line is defective in repairing IR-generated DSBs, we performed a neutral Comet assay. As shown in Figure 2B, the −/− and KD/− cell lines have significantly more unrepaired IR-induced DSBs compared to +/+ and +/− cells, as monitored by Comet tail moment. Next, we assessed the resolution of IR-induced 53BP1 foci in G_1_ phase of the cell cycle, which was used as a marker for DSB repair via the NHEJ pathway (23). 53BP1 foci resolution was markedly attenuated in the KD/− and −/− cell lines compared to the +/+ and +/− cell lines, demonstrating that the repair of IR-induced DSBs is impaired when the catalytic activity of DNA-PK_cs_ is lost (Figure 2C). The efficiency of NHEJ was also monitored *in vivo* via a GFP reporter assay. The −/− and KD/− cells showed a marked decrease in NHEJ-mediated DSB repair compared to the +/+ and +/− cell lines (Figure 2D). At last, utilizing a HR GFP reporter assay, we found that the −/− cell line has significantly increased HR frequency compared to the +/+, +/−, and KD/− cell lines (Figure 2E). The KD/− cell line showed an intermediate result, with increased HR efficiency in comparison to the +/+ and +/− cell lines, but decreased compared to the −/− cells (Figure 2E). Collectively, the data illustrate that inactivation of DNA-PK_cs_ catalytic activity results in decreased cell survival and DSB repair capacity in response to IR exposure.

### DNA-PK_cs_ initiates phosphorylation of H2AX and KAP1 at early time points following irradiation

**Figure 3.**
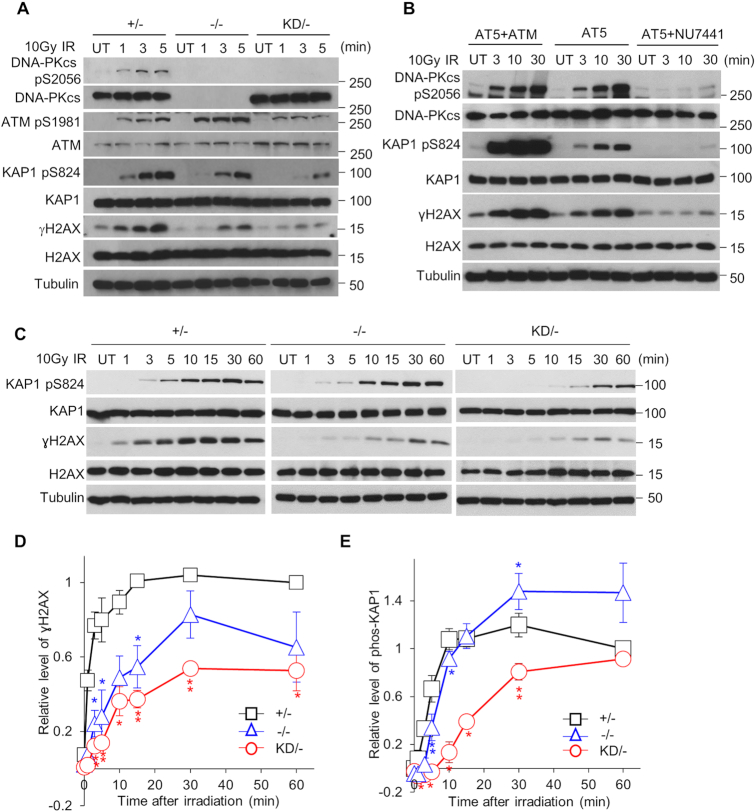
DNA-PK_cs_ initiates phosphorylation of H2AX and KAP1 after IR. (**A**) IR-induced phosphorylation of KAP1 and H2AX is attenuated in the KD/− cell line at early time points. HCT116 DNA-PK_cs_ +/−, −/−, and KD/− cell lines were mock-treated or irradiated with a dose of 10 Gy and allowed to recover for 1, 3, or 5 min. Whole cell lysates were obtained and immunoblotting was performed to assess the phosphorylation status of DNA-PK_cs_ at serine 2056, ATM at serine 1981, KAP1 at serine 824, and H2AX at serine 139. Tubulin was used as a loading control. (**B**) H2AX and KAP1 are phosphorylated in ATM-deficient cells (AT5) after IR, which is dependent on DNA-PK_cs_. AT5 cells were mock-treated or incubated for 2 h prior to irradiation with 10 μM NU7441 to inhibit DNA-PK_cs_ and then AT5 cells and ATM-expressing AT5 cells (AT5+ATM) were mock-treated or irradiated with a dose of 10 Gy and allowed to recover for 3, 10, or 30 min. Whole cell lysates were obtained and immunoblotting was performed to assess the phosphorylation status of DNA-PK_cs_ at serine 2056, KAP1 at serine 824, and H2AX at serine 139. Tubulin was used as a loading control. (**C**–**E**) Time courses of IR-induced KAP1 and H2AX phosphorylation in the HCT116 DNA-PK_cs_ +/−, −/−, and KD/− cell lines. The cells were mock-treated or irradiated with a dose of 10 Gy and allowed to recover for 1, 3, 5, 10, 15, 30, or 60 min. (C) Whole cell lysates were obtained and immunoblotting was performed to assess the phosphorylation status of DNA-PK_cs_ at serine 2056, KAP1 at serine 824, and H2AX at serine 139. Tubulin was used as a loading control. All immunoblots for each protein shown in (C) come from the same exposure of a single film. The relative phosphorylation level of H2AX and KAP1 at each time point are shown in (D) and (E), respectively. Following quantification of the protein levels, the relative level of H2AX and KAP1 phosphorylation were calculated by taking γH2AX/H2AX and KAP1 S824/KAP1 and then normalizing each time point to value of γH2AX/H2AX and KAP1 S824/KAP1 at 1 h after IR in the (+/−) control cell line. The data are presented as the mean ± SEM from three independent experiments. The *P*-values were generated by comparing +/− with −/− or KD/− with Student *t*-test. *, *P*< 0.05; **, *P* < 0.01. All data (A–C) are a representative image of three independent experiments.

Multiple studies have found that the DNA–PK complex is recruited to DSBs immediately after their induction (16–18), which drove us to hypothesize that DNA-PK_cs_ activity may be responsible for the initial phosphorylation events at chromatin after IR-mediated DSBs. To test this hypothesis, we examined the ability of DNA-PK_cs_ kinase activity to influence DDR signaling at early time points (1, 3, and 5 min) post-IR (10 Gy of γ-rays) by focusing on phosphorylation events on chromatin, including phosphorylation of the histone variant H2AX at serine 139, termed γH2AX (31) and the chromatin remodeling factor KRAB domain-associated protein 1 (KAP1) at serine 824 (KAP1 pS824) (32). Since the +/− cell line has similar DSB repair capacity as the +/+ cell line and has the same number of DNA-PK_cs_-expressing alleles as the KD/− cell line, we utilized the +/− cell line as the control wild-type cell line for the rest of the study. DNA-PK_cs_ was activated, as monitored by autophosphorylation of DNA-PK_cs_ at S2056 (pS2056), within 60 s of DSB induction in the +/− cell line, but not in the −/− and KD/− cell lines (Figure 3A). A marked decrease in H2AX and KAP1 phosphorylation at these early time points post-IR was observed in the KD/− cells compared to the +/− cells, with the −/− cells showing an intermediate response (Figure 3A). A similar decrease in H2AX and KAP1 phosphorylation was also observed in the −/− and KD/− cells after treatment with 2 and 5 Gy of IR (Supplementary Figure S2A). Pre-treating cells with the DNA-PK_cs_ inhibitor NU7441 also resulted in a decrease in IR-induced γH2AX and KAP pS824 at early time points post-IR in the HCT116 +/+, U2OS, and 1BR3 cell lines (Supplementary Figure S2B–D), which supports the model that DNA-PK_cs_ is responsible for the early phosphorylation of KAP1 and H2AX after DNA damage. It should be noted, the decrease in γH2AX and KAP1 pS824 was not due to defects in the MRN complex or ATM, as the levels of all four of these proteins were equivalent in the +/−, −/−, and KD/− cell lines (Supplementary Figure S2E). ATM was also activated by IR, as monitored by the autophosphorylation of ATM at S1981 (ATM p1981), in all three cell lines at these early time points, but phospho-ATM focus formation was attenuated in the KD, compared to the +/− and −/− cells (Supplementary Figure S3). To verify that DNA-PK_cs_ phosphorylates H2AX and KAP1 independently of ATM, we examined IR-induced γH2AX and KAP1 pS824 formation in the ATM-defective cell line AT5BIVA (AT5). H2AX and KAP1 were phosphorylated at early time points in response to IR in AT5 cells, and these phosphorylation events were abrogated when DNA-PK_cs_ was inhibited via pre-treatment with NU7441 (Figure 3B and Supplementary Figure S2D). At last, a time course following IR treatment showed that H2AX phosphorylation was attenuated from 1 to 60 min and KAP1 phosphorylation from 1 to 30 min in KD/− cells compared to +/− cells (Figure 3C–E). Collectively, the data paint a compelling picture that DNA-PK_cs_ is the initial kinase responsible for phosphorylating KAP1 and H2AX following IR-induced DSB formation.

### DNA-PK_cs_ catalytic activity is required for chromatin relaxation immediately after DNA DSB induction

**Figure 4.**
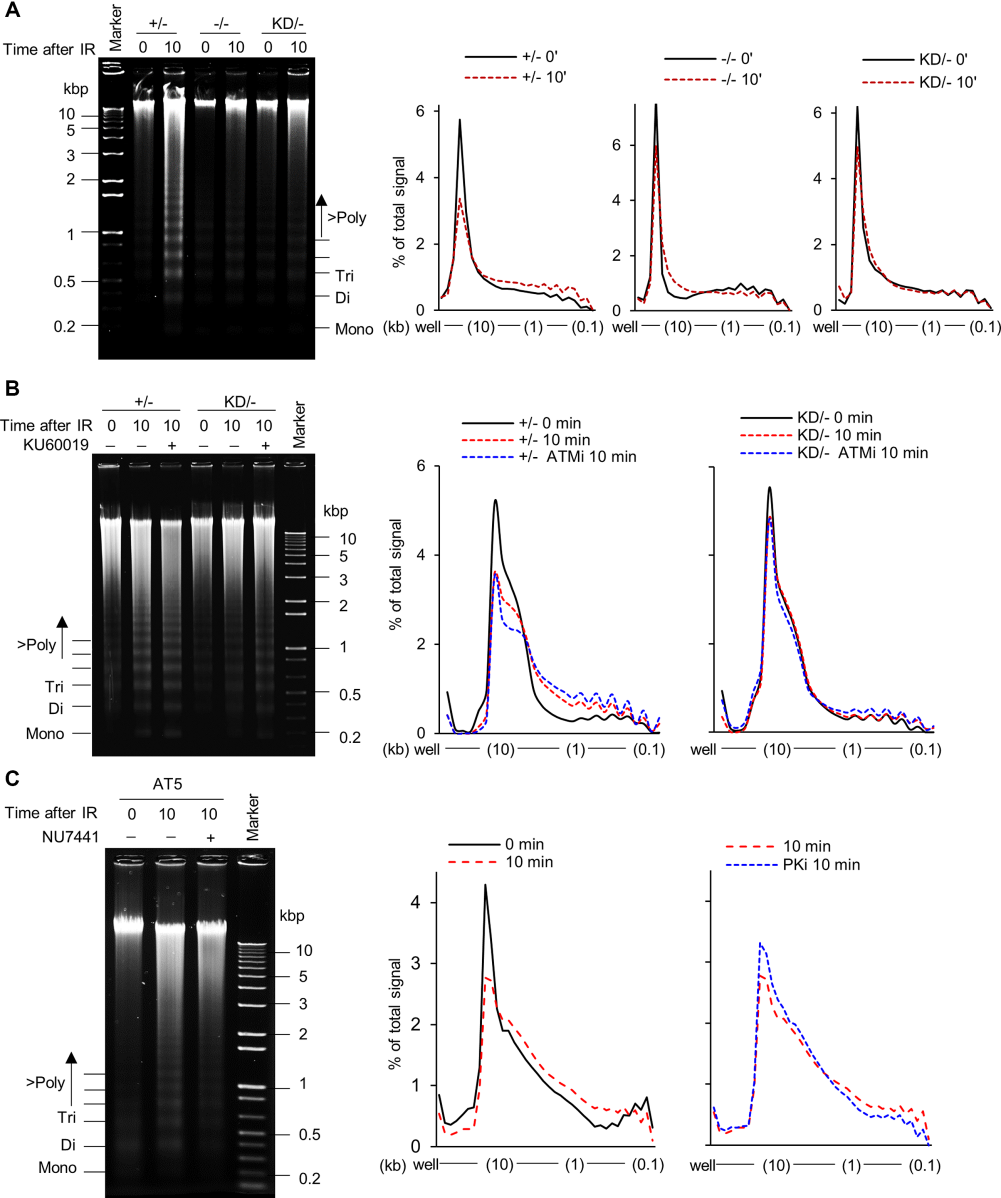
Chromatin decondensation in response to DNA damage at early time points requires DNA-PK_cs_ kinase activity. (**A**) IR-induced chromatin relaxation is attenuated in the HCT116 DNA-PK_cs_ KD/− and −/− cells. The HCT116 DNA-PK_cs_ +/−, −/−, and KD/− cell lines were mock-treated or irradiated with a dose of 10 Gy and allowed to recover for 10 min. Chromatin decondensation was then determined by examining micronuclease (MNase) accessibility. Nuclei were processed and the DNA was visualized by resolving it via agarose gel electrophoresis. Panels on the right show quantified signal as the percent of total for each lane across a distance from the well to the end of gel. (**B**) ATM kinase activity is not required for the initial chromatin relaxation after IR-induced DNA damage. The HCT116 DNA-PK_cs_ +/− and KD/− cell lines were mock-treated or incubated for 2 h prior to irradiation with 10 μM KU60019 to inhibit ATM and then the cells were mock-treated or irradiated with a dose of 10 Gy and allowed to recover for 10 min. Samples were processed and quantified as described in (A). (**C**) Inhibition of DNA-PK_cs_ suppresses IR-induced chromatin decondensation in ATM-deficient AT5 cells. The AT5 cells were mock-treated or incubated for 2 h prior to irradiation with 10 μM NU7441 to inhibit DNA-PK_cs_ and then the cells were mock-treated or irradiated with a dose of 10 Gy and allowed to recover for 10 min. Samples were processed and quantified as described in (A). All data (A–C) are a representative image of three independent experiments.

Chromatin relaxation/decondensation in response to DSBs has been reported to be mediated by both KAP1 and H2AX phosphorylation (32,33). Since DNA-PK_cs_ phosphorylates KAP1 and H2AX immediately after DSB induction, it prompted us to examine if DNA-PK_cs_ is required for the initial remodeling of chromatin after DNA damage. To this end, chromatin digestion via micrococcal nuclease (MNase) treatment was monitored post-irradiation. MNase-mediated digestion is increased concomitantly with increased accessibility of the chromatin and the linker region between nucleosomes due to chromatin decondensation following DNA damage (32). IR-induced DNA damage resulted in a marked increase in chromatin accessibility to MNase in the +/− cell line 10 min after IR, but this was markedly attenuated in the −/− and KD/− cell lines, suggesting that DNA-PK_cs_ kinase activity is required for chromatin remodeling following DSB induction (Figure 4A). Further evidence for DNA-PK_cs_ modulating chromatin decondensation following DNA damage was obtained using the radiomimetic agent, neocarzinostatin (NCS). NCS treatment resulted in increased KAP1 and H2AX phosphorylation and chromatin relaxation in the +/− cell line, but not in the KD/− cell line (Supplementary Figure S4A and B). Chromatin relaxation was not affected by pre-treatment with the ATM inhibitor KU60019 in the +/− and KD/− cell lines, which further supports the model that IR-induced chromatin decondensation at early time points is mediated by DNA-PK_cs_ kinase activity and not ATM (Figure 4B and Supplementary Figure S5A). In line with this observation, chromatin decondensation occurred in ATM-deficient AT5 cells, and this was attenuated when the cells were pre-treated with the DNA-PK_cs_ inhibitor, NU7441 (Figure 4C and Supplementary Figure S5B). Taken together, these results imply that DNA-PK_cs_ catalytic activity is required for chromatin decondensation at early time points following DNA damage.

### DNA-PK_cs_ enzymatic activity promotes the rapid recruitment of DDR proteins to DSB sites

**Figure 5.**
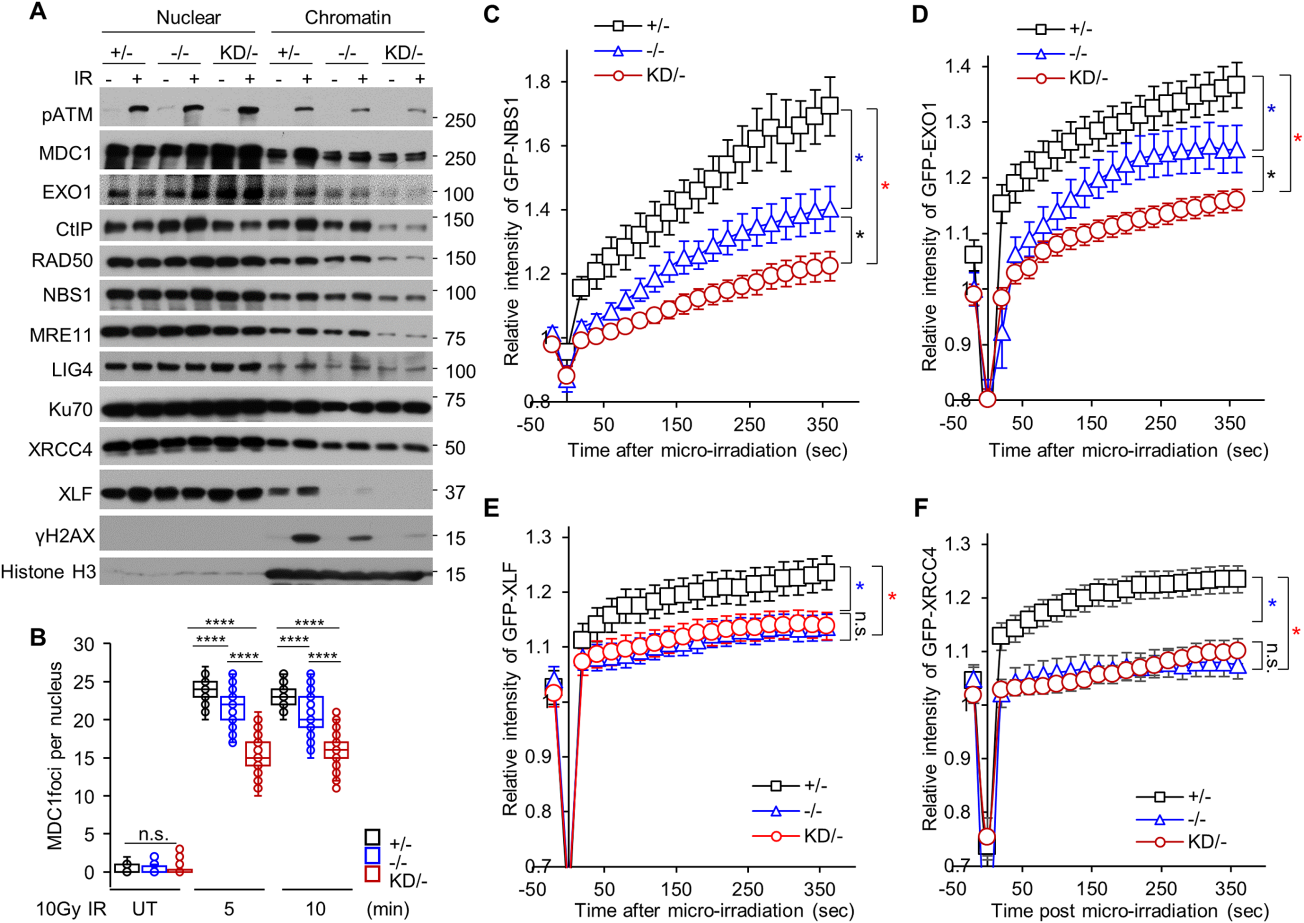
DNA-PK_cs_ catalytic activity facilitates the initial recruitment of the DDR machinery to DSBs. (**A**) Recruitment of HR and NHEJ factors to the chromatin after IR is attenuated in the HCT116 DNA-PK_cs_ KD/− cell line. HCT116 DNA-PK_cs_ +/−, −/−, and KD/− cells were mock-treated or irradiated with a dose of 10 Gy and allowed to recover for 10 min. Subsequently, soluble nuclear and the chromatin-enriched fractions were isolated for immunoblotting to assess the recruitment of the proteins listed in the figure to the chromatin after irradiation. (**B**) IR-induced focus formation of MDC1 is attenuated in the HCT116 DNA-PK_cs_ KD/− cell line. The HCT116 DNA-PK_cs_ +/−, −/−, and KD/− cell lines were mock-treated or irradiated with a dose of 1 Gy and MDC1 foci formation was assessed 5 and 10 min later. MDC1 focus formation was examined in at least 50 cells and the number of MDC1 IR-induced foci per nucleus is shown. ****, *P* value < 0.0001. (**C**–**F**) Recruitment of GFP-tagged (C) NBS1, (D) EXO1, (E) XLF, and (F) XRCC4 to laser-generated DSBs is attenuated in the HCT116 DNA-PK_cs_ KD/− cell line compared to the +/− cells. Relative fluorescent intensity of GFP-tagged NBS1, EXO1, XLF, and XRCC4 are presented as the mean ± SEM. *, *P* value < 0.05.

Chromatin remodeling makes the area around a DNA lesion more accessible for DNA damage response and repair proteins (34). We hypothesized that the initial local chromatin relaxation induced by DNA-PK_cs_ catalytic activity is required for the rapid loading of the DDR machinery to DSBs. To examine this, chromatin fractions were isolated 10 min pre- or post-DNA damage in +/−, −/−, and KD/− cells. After DSB induction, MRE11, NBS1, RAD50, Mediator of DNA Damage Checkpoint protein 1 (MDC1), C-Terminal Interacting protein 1 (CtIP), and Exonuclease 1 (EXO1) all showed increased recruitment to the chromatin fraction in the +/− cell line following IR exposure, illustrating that each of these proteins are quickly recruited to DNA following damage (Figure 5A). In striking contrast, recruitment of each of these factors to chromatin was attenuated in the KD/− and −/− cell lines, suggesting that DNA-PK_cs_ kinase activity is required for the recruitment and/or loading of the DDR machinery to damaged DNA (Figure 5A). Immunofluorescence studies also showed that IR-induced MDC1 (Figure 5B and Supplementary Figure S6A), γH2AX (Supplementary Figure S6B), and phospho-ATM (Supplementary Figure S3) focus formation was attenuated in the KD/− and −/− cell lines at early time points post-irradiation compared to the +/− cell line. The hypothesis that recruitment of DDR factors to DSBs requires DNA-PK_cs_ kinase activity was additionally supported by data showing that the localization of GFP-tagged NBS1 (Figure 5C and Supplementary Figure S6C) and EXO1 (Figure 5D and Supplementary Figure S6D) to laser-generated DSBs was attenuated in the KD/− and the −/− cell lines compared to the +/− cell line. To enhance this analysis, we examined if DNA-PK_cs_ catalytic activity modulated the recruitment of the NHEJ machinery to DSBs. The localization of the Ku heterodimer to IR-induced (Figure 5A) and laser-generated DSBs (Supplementary Figure S6E) was not affected in the KD/− cells. However, recruitment of DNA Ligase IV (LIG4), X-Ray Cross Complementing Group 4 protein (XRCC4), and XRCC4-Like Factor (XLF) to chromatin fractions following IR treatment was attenuated in the KD/− and −/− cell lines compared to the +/− cell line (Figure 5A). Furthermore, recruitment of GFP-tagged XLF (Figure 5E and Supplementary Figure S6F) and XRCC4 (Figure 5F and Supplementary Figure S6G) to laser-induced DSBs was decreased in the KD/− and −/− cell lines compared to the +/− cell line. Finally, the recruitment of GFP-NBS1 to laser-induced DSBs is not disrupted in the DNA Ligase 4 null HCT116 (LIG4 −/−) (35) cell line compared to the recruitment in the +/+ cell line (Supplementary Figure S7). This result indicates that the decrease in the recruitment of the DDR machinery to DSBs in the KD/− cell line is caused by the loss of DNA-PK_cs_ catalytic activity and not simply due to a loss in NHEJ capacity. Collectively, these data support the notion that DNA-PK_cs_ catalytic activity is required for the rapid recruitment of the DDR machinery to DSBs.

## DISCUSSION

The enzymatic activity of DNA-PK_cs_ plays a critical function in the cellular response to DSBs, but its role(s) in this process has mostly been constrained to the logistical aspects of physically mediating DSB repair via the NHEJ pathway and tethering and/or protecting DSB ends from non-specific processing. In this report, using a human cell line in which a point mutation was introduced to inactivate the kinase domain of DNA-PK_cs_ (KD/−), we demonstrate that immediately after DSB induction DNA-PK_cs_-mediates phosphorylation of H2AX and KAP1, promotes chromatin decondensation, and is required for the rapid recruitment of the DDR machinery to sites of the DNA damage. These observations identify DNA-PK as the kinase responsible for the initial phosphorylation events at damaged chromatin, and also reveal an underlying mechanism responsible for the programed cellular response to DSBs in human cells.

The DSB sensors, the Ku70/80 heterodimer and the MRN complex, recognize DNA damage and recruit and activate the protein kinases DNA-PK_cs_ and ATM, respectively, to damaged chromatin, in order to facilitate efficient signaling and repair of DSBs (4,6,36). The role of ATM kinase activity in the DDR is clearly defined, as ATM phosphorylates hundreds of substrates in order to initiate signaling cascades, modulate the cell cycle, regulate chromatin remodeling, and facilitate a subset of DSB repair (8). In contrast, the role of DNA-PK_cs_ catalytic activity in the DDR is much less well characterized. Experimentally, this has been due principally to the functional redundancy between ATM, ATR, and DNA-PK_cs_. Previous researchers have utilized virtually exclusively DNA-PK_cs_-null cell lines to assess the functional consequences of the loss-of-function of DNA-PK_cs_ kinase activity (20,29,37–39). In the complete absence of DNA-PK_cs_, however, the functional redundancy provided by ATM and ATR has made the ensuing analyses of such DNA-PK_cs_-null cell lines equivocal. To overcome this experimental hurdle, we constructed a human cell line that still expresses DNA-PK_cs_ protein, but one that is functionally inactive. In this context, we believe that the inactive DNA-PK_cs_ cannot be complemented by ATM and ATR, because the DSB ends will not be free as they are in the DNA-PK_cs_ null cells and thus ATM and ATR are not as readily activated at the DSB site. Furthermore, it is probable that the kinase dead DNA-PKcs protein will occlude the DNA ends due to decreased dissociation kinetics from DSB ends. Studies using analogously engineered hamster cells (16,40) and mice (30,41) have come to a similar conclusion. Here, we have utilized our kinase-dead cells to identify—for the first time—that DNA-PK_cs_ plays an initial role in mediating the phosphorylation events immediately after DSB induction. We postulate that the DNA–PK complex is the first responder to DSBs and that DNA-PK_cs_ catalytic activity initiates the DDR in human cells. This hypothesis is supported by spatiotemporal studies, which showed that Ku/DNA-PK_cs_ are directly recruited to the sites of DSBs within seconds of their creation and—importantly—are recruited to DSB sites faster than MRN/ATM (16–18). Notably, both the Ku heterodimer and DNA-PK_cs_ are highly abundant (at least 5 × 10^5^ molecules per cell) in human cells. Moreover, Ku70/80 has an extremely high affinity for DSB ends in a sequence-independent manner (42). All these features endow the DNA–PK complex as the naturally designed ‘first responder’ to DSBs in human cells.

In eukaryotic cells, DNA is complexed with histones to form chromatin, which is a highly dynamic structure that packages the genome into the confines of the nucleus and regulates the accessibility of the DNA for transcription, replication, and DSB repair (34). Two key modifications that generate a chromatin state permissive for repair and which directly contribute to the recruitment of the DDR machinery to a DSB are phosphorylations of the histone variant H2AX and KAP1 (32,33). H2AX is phosphorylated rapidly (within seconds) after DSB induction and this mark serves as a platform for the recruitment of additional DDR factors at the site of DNA damage (43). MDC1, the major reader of the resulting phospho-H2AX mark (i.e. γH2AX), is recruited by γH2AX, and it, in turn, recruits MRN and ATM to the regions surrounding the damaged sites. The recruitment of MRN and ATM results in more H2AX phosphorylation, which results in propagation of γH2AX over several megabases surrounding the break (44). Multiple studies have reported that H2AX is phosphorylated by ATM, with DNA-PK_cs_ likely playing a redundant role in this process (15,45–47). However, in this report, we show that DNA-PK_cs_ catalytic activity is required for the rapid and efficient phosphorylation of H2AX in response to IR-mediated DSBs. That DNA-PK_cs_ is playing an active, and not passive or back-up, role in phosphorylating H2AX is further supported by two lines of evidence. First, H2AX is phosphorylated in ATM-deficient cells, albeit at lower levels than when compared to ATM-proficient cells, in response to IR-induced DSBs and this phosphorylation is mediated by DNA-PK_cs_ (Figure 3B). Second, deletion of ATM reduces the extent of the γH2AX domain, but—again importantly—does not abolish H2AX phosphorylation in close proximity to the DSB (48). Collectively, these data suggest a model in which DNA-PK_cs_ is recruited rapidly and directly to the DSB site and is responsible for the initial phosphorylation of H2AX in close proximity to the DSB. This hypothesis is also supported by *in vitro* biochemical data that showed that DNA-PK can be activated by nucleosomes with a short free end (20 to 30 bp) of DNA and that DNA-PK_cs_ can subsequently phosphorylate H2AX within the nucleosome (49). Furthermore, the decrease in γH2AX induction in the DNA-PK_cs_ KD/− cell line results in a concomitant decrease in MDC1, MRN, and phospho-ATM accumulation at IR-induced DSBs at early time points. Collectively, our results indicate that DNA-PK_cs_ catalytic activity plays an active role in initiating the creation of the γH2AX domain by directly phosphorylating H2AX near the DSB site, and also by modulating the amplification of the γH2AX signal by its ability to influence, both directly and indirectly, the recruitment and potentially the fast activation of ATM at chromatin.

As stated above, another early event in the DDR is phosphorylation of KAP1, which is required for chromatin relaxation. Similar to H2AX, multiple studies concluded that KAP1 is phosphorylated by ATM, with DNA-PK_cs_ regarded as a likely redundant kinase (50). However, our data show that at early time points post-irradiation that DNA-PK_cs_ is the kinase responsible for phosphorylating KAP1. Akin to what we proposed above for H2AX, we postulate that DNA-PK_cs_ phosphorylates KAP1 proximal to the DSB site whereas ATM phosphorylates it distally. This model is supported by a study showing that treatment of irradiated cells with the DNA-PK_cs_ inhibitor NU7026 largely abolished phosphorylation of KAP1 at laser-generated DSBs, but did not affect pan‐nuclear KAP1 phosphorylation, which was found to be mediated by ATM (15). Mechanistically, we propose that DNA-PK_cs_-mediated phosphorylation of KAP1 results in chromatin decondensation immediately after IR-induced DSBs, which we believe occurs proximal to the DSB site. However, it should be noted that the extent of chromatin relaxation in relation with the position of the DSB still needs to be addressed by high-resolution experiments. This local chromatin relaxation is required for the subsequent rapid recruitment of additional DDR machinery at the DSB site, including the factors required for NHEJ, HR, and DDR signaling. In summary, we postulate that DNA-PK_cs_ directly phosphorylates H2AX and KAP1 near the DSB site, but that it also influences their phosphorylation by assisting in the recruitment of the MRN complex and ATM to DSBs after chromatin decondensation near the DSB site.

Collectively, our results support a model in which there is spatiotemporal regulation of the DDR at IR-generated DSBs (Supplementary Figure S8). Immediately (literally within seconds) after the induction of a DSB, the Ku heterodimer recognizes and localizes to the DNA lesion, where it recruits DNA-PK_cs_. DNA-PK_cs_ subsequently phosphorylates H2AX and KAP1 in the immediate vicinity of the DSB site. This phosphorylation of H2AX and KAP1 results in local chromatin relaxation, which allows the rapid recruitment of the rest of the DDR machinery, including ATM, at and/or near the DSB site. ATM continues to phosphorylate H2AX and KAP1 distal from the site of the DSB. Thus, ATM’s role is to amplify the signal generated by DNA-PK_cs_ in response to the DSB. Mechanistically, this includes phosphorylation of H2AX over several megabases near and distal from the DSB site as well as phosphorylation of KAP1, which results in extended chromatin decondensation. This biphasic response allows for rapid and regulated induction of the DDR. We postulate that the main reason that DNA-PK_cs_’ role in the initial response in the DDR has been missed is due to the fact that it simply initiates the signal and is not required for its amplification, which is much greater; this feature is enhanced by the redundancy of the PIKKs, which allows them to compensate for the loss of each other.

## Supplementary Material

gkz694_Supplemental_File
