# Supplementary material for: DNA-PKcs promotes chromatin decondensation to facilitate initiation of the DNA damage response
Source: Nucleic Acids Res. 2019 Aug 9;47(18):9467–79. doi: 10.1093/nar/gkz694 (PMC6765147; doi:10.1093/nar/gkz694)
Supplement: gkz694_Supplemental_File [file gkz694_supplemental_file.pdf]

Supplemental materials

DNA-PK$_{cs}$ promotes chromatin decondensation to facilitate initiation of the DNA damage response

Huiming Lu[1], Janapriya Saha[1], Pauline J. Beckmann[2], Eric Hendrickson[2], and Anthony J. Davis[1*]

[1] Division of Molecular Radiation Biology, Department of Radiation Oncology, UT Southwestern Medical Center, Dallas, TX, 75390, USA

[2] Department of Biochemistry, Molecular Biology, and Biophysics, University of Minnesota Medical School, Minneapolis, MN, 55455, USA

*Correspondence: anthony.davis@utsouthwestern.edu

Supplemental Table 1. Primers used in rAAV synthesis and cell line validation.

Primer name	Sequence 5' --> 3'
Construction of conditional exon 79 vector	
PKcs79LArmF	ATACATACGCGGCCGCGTCAGTTCCCTTTAAGTGAAAACTTTTCAGTAAAAGG
PKcs79LArmR	GCTCCAGCTTTTGTTCCCTTTAGCTTAAATAGAACTGTTGTTCAAACACTGGAGAC
PKcs79RArmF	CGCCCTATAGTGAGTCGTATTACGCTGTGTGTGAGTATCCACTATAAAATATGTGTAGC
PKcs79LoxPR	GTATAATGTATGCTATACGAAGTTATCTACTGGCTGGGAGCAGCCTGGC
PKcs79LoxPF	TCGTATAGCATACATTATACGAAGTTATTTTTCTAGTGAAGAAAGAGCAGTTTGG
PKcs79RArmR	ATACATACGCGGCCGCCCTTCAACAATTTTTCTTCCCCCCACCCAAAG
Additional primers for KD vector construction	
PKcs79KDMutF	TCCTGGTGAgGGGTGGCGAGGACCTGCGGCAGGACCA
PKcs79KDMutR	CTCGCCACCCcTCACCAGGAAAGGGTGTTCCCTCTCGTCAT
Validation of correct vector targeting	
PKcs79_EF1	CTTCACCATGTAAGCCAGGATGGTCTCTG
PKCS79_ER	GTTAAAAATTGAGAACAGTACCTAGCCCACAG

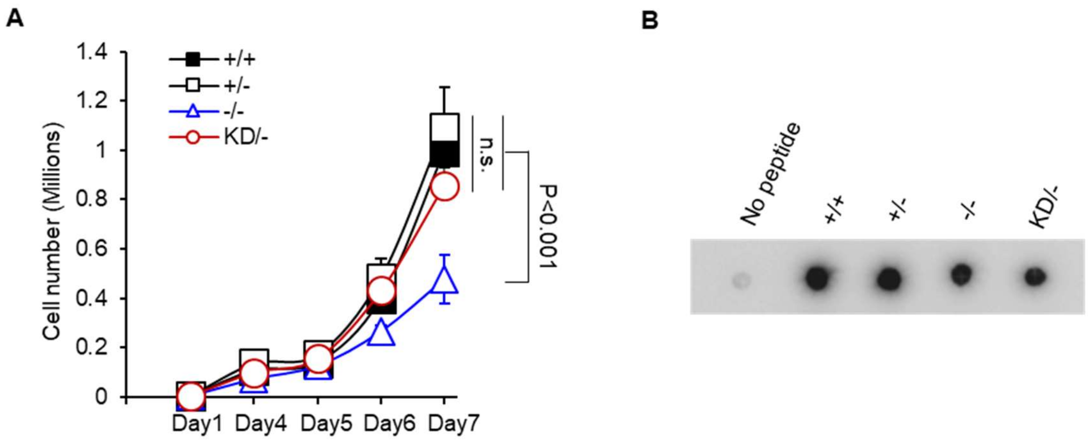

Supplemental Fig. 1. Characterization of HCT116 DNA-PK$_{cs}$ kinase-dead cells. **(A)** Cell proliferation of HCT116 DNA-PK$_{cs}$ +/+, +/-, -/- and KD/-. **(B)** In vitro kinase activity of the nuclear extract from HCT116 DNA-PK$_{cs}$ +/+, +/-, -/- and KD/- cells on biotin-labelled H2AX peptide.

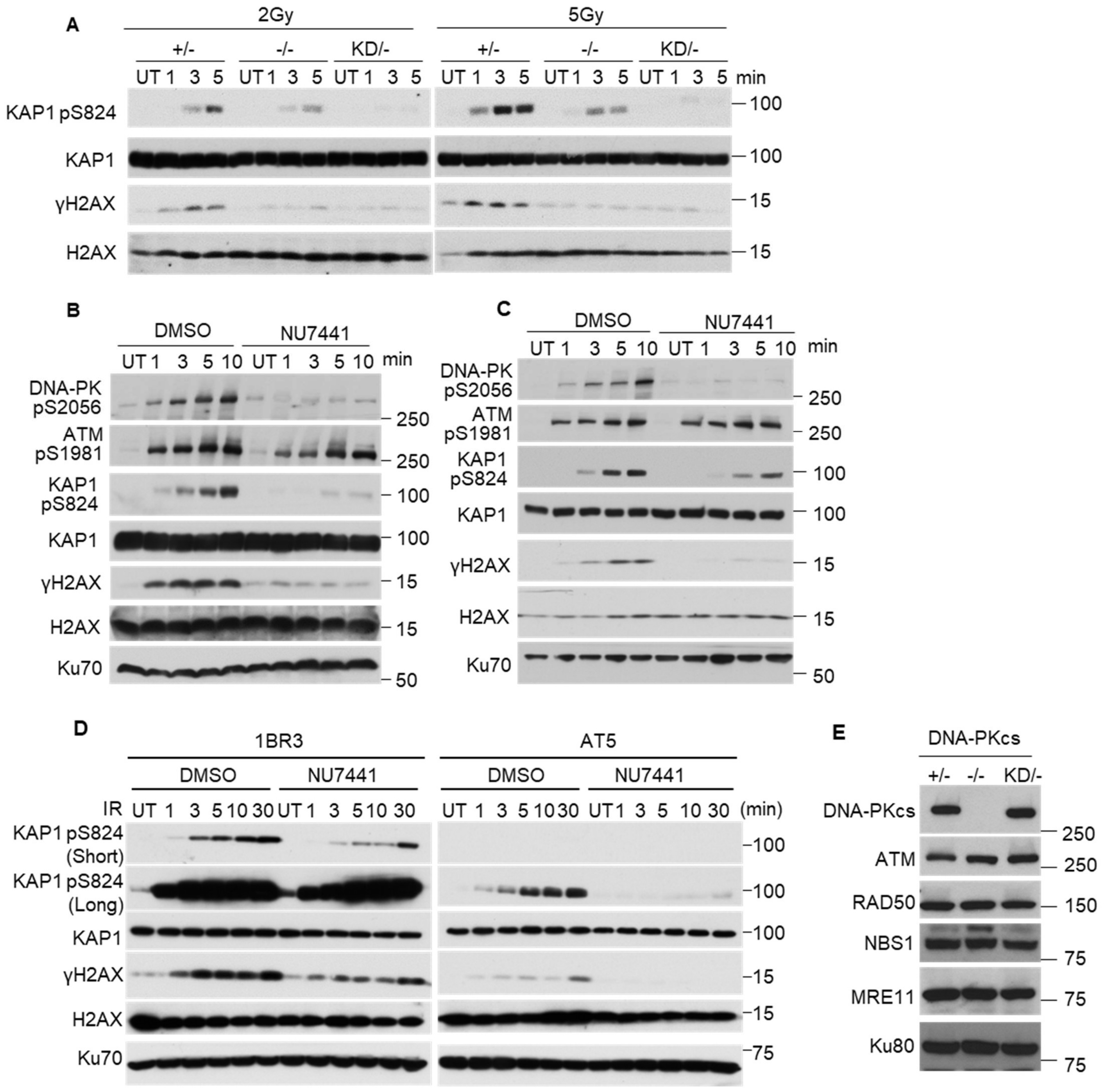

Supplemental Fig. 2. DNA-PK$_{cs}$ kinase is important for phosphorylation of H2AX and KAP1 at Ser139 and Ser824, respectively, at the early phase of DNA damage response to IR. **(A)** Phosphorylation of H2AX and KAP1 were inhibited in HCT116 DNA-PKcs -/- and KD/- cells after IR with a dose of 2 Gy and 5 Gy. **(B-D)** IR-induced phosphorylation of H2AX at Ser139 (γH2AX) and KAP1 at Ser834 is attenuated when cells are pretreated with the DNA-PK$_{cs}$ inhibitor NU7441. **(B)** HCT116 +/+ cells, **(C)** U2OS, and **(D)** 1BR3 cells were mock-treated (DMSO treated) or incubated for 2 hr prior to irradiation with 20 μM

NU7441 and then the cells were mock-treated or irradiated with a dose of 10 Gy and allowed to recover for 1, 3, 5, or 10 min. Whole cell lysates were obtained and immunoblotting was performed to assess phosphorylation of DNA-PKcs at serine 2056, ATM at serine 1981, KAP1 at serine 824, and H2AX at serine 139. Immunoblotting of Ku70 was used as a loading control. **(E)** Expression levels of MRE11-RAD50-NBS1 complex and ATM are not affected by inactivation of DNA-PK_{cs} in HCT116 cells. Protein levels of MRE11, RAD50, NBS1, and ATM were measured in HCT116 DNA-PK_{cs} +/-, -/- and KD/- cells by Western blotting.

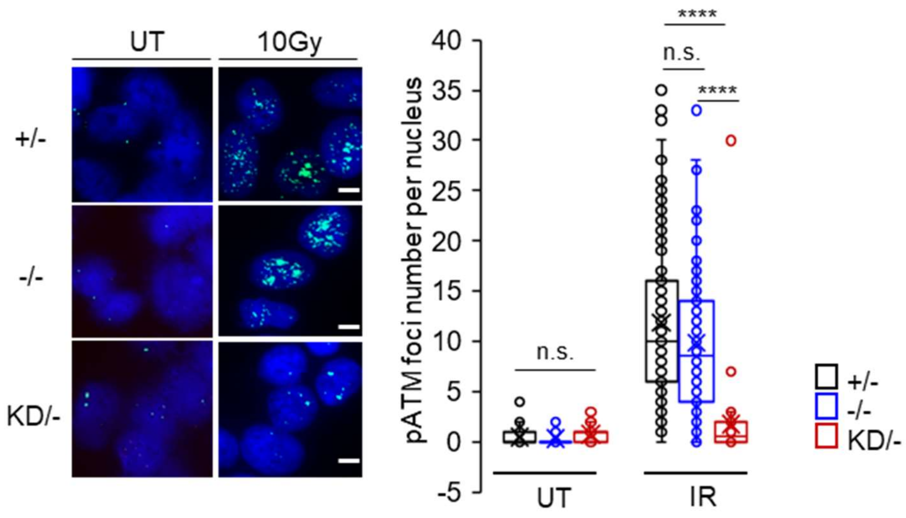

Supplemental Fig. 3. IR-induced focus formation of phosphorylated ATM at S1981 is attenuated in the DNA-PK$_{cs}$ KD/- cell line. The HCT116 DNA-PK$_{cs}$ +/-, -/-, and KD/- cell lines were mock-treated or irradiated with a dose of 10 Gy and phospho-ATM S1981 foci formation was assessed 5 and 10 min later. After the recovery from the irradiation, the cells were pre-extracted with CSK buffer containing 10mM HEPES 7.4, 300mM sucrose, 100mM NaCl, 3mM $MgCl_2$ and 0.1% Triton X100. After fixing with 3.7% PFA, the cells were then processed for immunofluorescence staining with the antibody against phospho-ATM S1981 ATM. Phospho-ATM S1981 focus formation was examined in at least 50 cells and the number of phospho-ATM S1981 IR-induced foci per nucleus is shown. ****, p value < 0.0001. Bar, 5 μm.

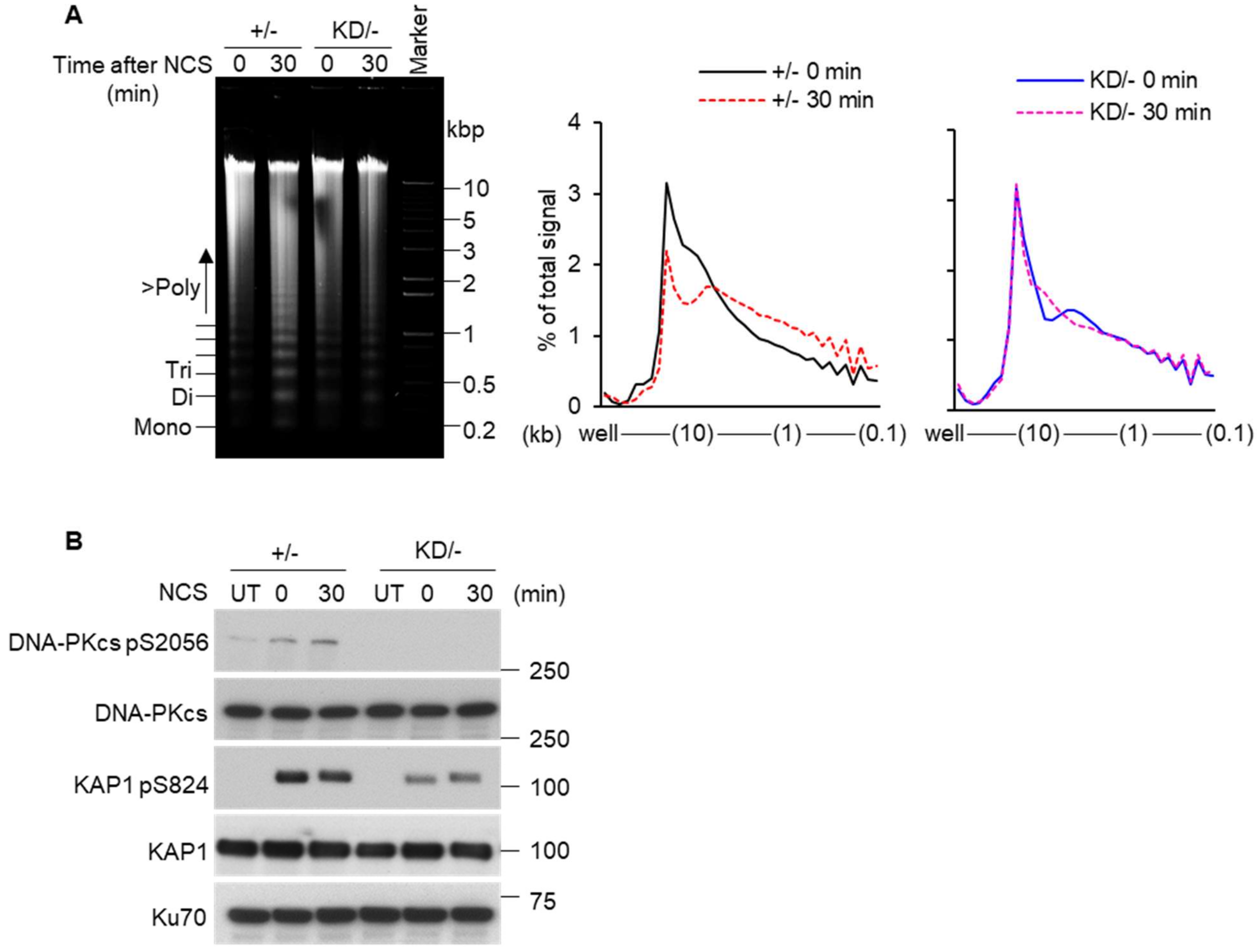

Supplemental Fig. 4. Chromatin relaxation is attenuated in HCT116 DNA-PK$_{cs}$ KD/- cells in response to treatment with the DNA damaging agent neocarzinostatin (NCS). **(A)** Chromatin opening was measured by MNase accessibility in +/- and KD/- cells after NCS treatment. The cells were mock treated or treated with 200 ng/mL NCS for 30 min and then MNase accessibility analysis was performed as outlined in the manuscript. The presented image is one of three independent experiments. Quantitation of nucleosome signal were presented as percent of total across distance from the well to end of the gel. **(B)** NCS-mediated KAP1 phosphorylation is attenuated in the DNA-PKcs KD/- cell line. HCT116 DNA-PKcs +/- and KD/- cells were mock treated or treated with 200 ng/mL NCS. The NCS was withdrawn and then the cells were harvested immediately after the withdrawal or allowed to incubate for 30 min. Whole cell lysates were obtained and immunoblotting was performed to assess phosphorylation of KAP1 at serine 824 and DNA-PKcs at serine 2056. Ku70 was used as a loading control.

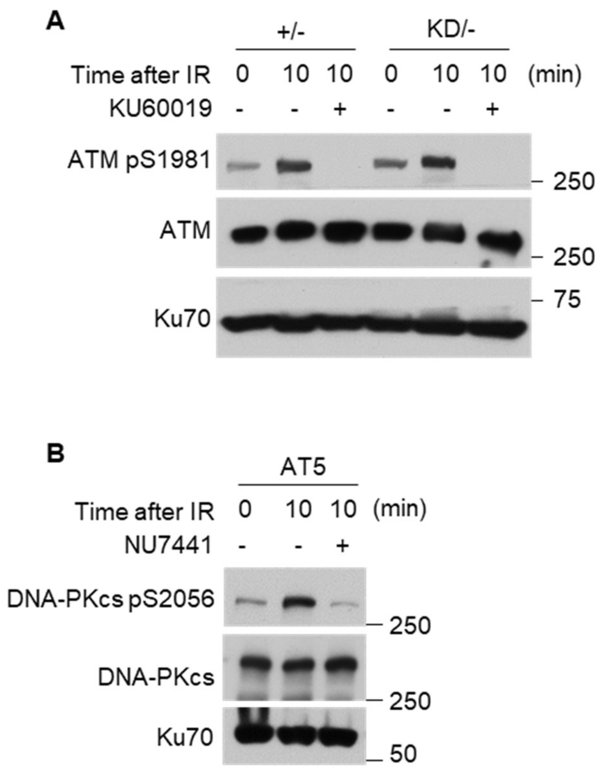

Supplemental Fig. 5 Pretreatment with KU60019 and NU7441 inhibits ATM and DNA-PK in **(A)** HCT116 and **(B)** AT5 cells, respectively. HCT116 DNA-PKcs +/- and KD cells were pretreated with 10 μM KU60019 for 2 hr before a 10Gy-γ-radiation. Cells were withdrawn immediately or 10 min after irradiation for immunoblotting analysis. For AT5 cells, 10 μM NU7441 was applied for DNA-PK inhibition. Inhibition of ATM by KU60019 and DNA-PK by NU7441 were examined with auto-phosphorylation level of these two kinases.

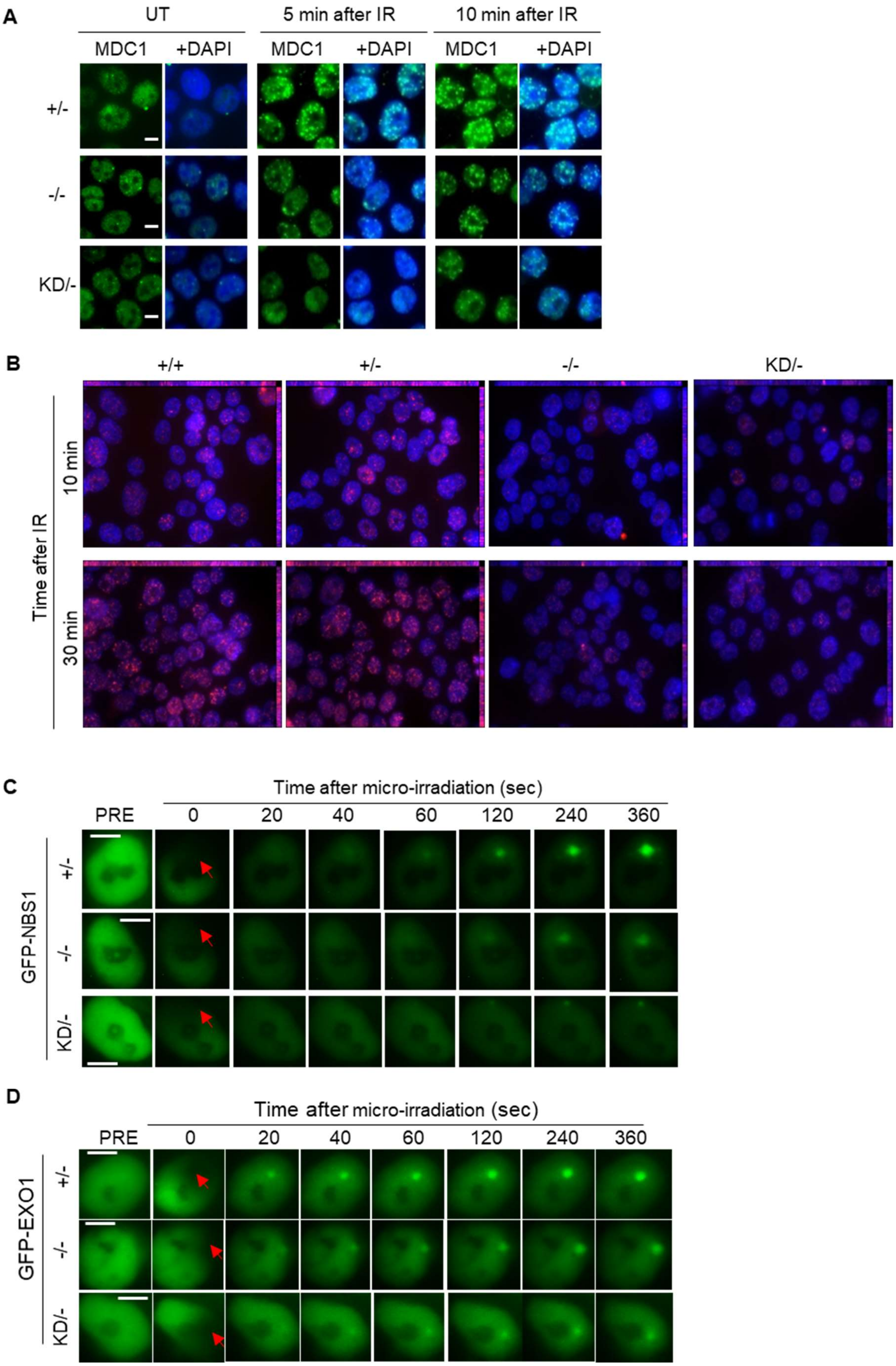
A
UT
5 min after IR
10 min after IR
MDC1
+DAPI
+/-
-/-
KD/-
B
+/+
+/-
-/-
KD/-
Time after IR
10 min
30 min
C
Time after micro-irradiation (sec)
PRE
0
20
40
60
120
240
360
GFP-NBS1
D
GFP-EXO1

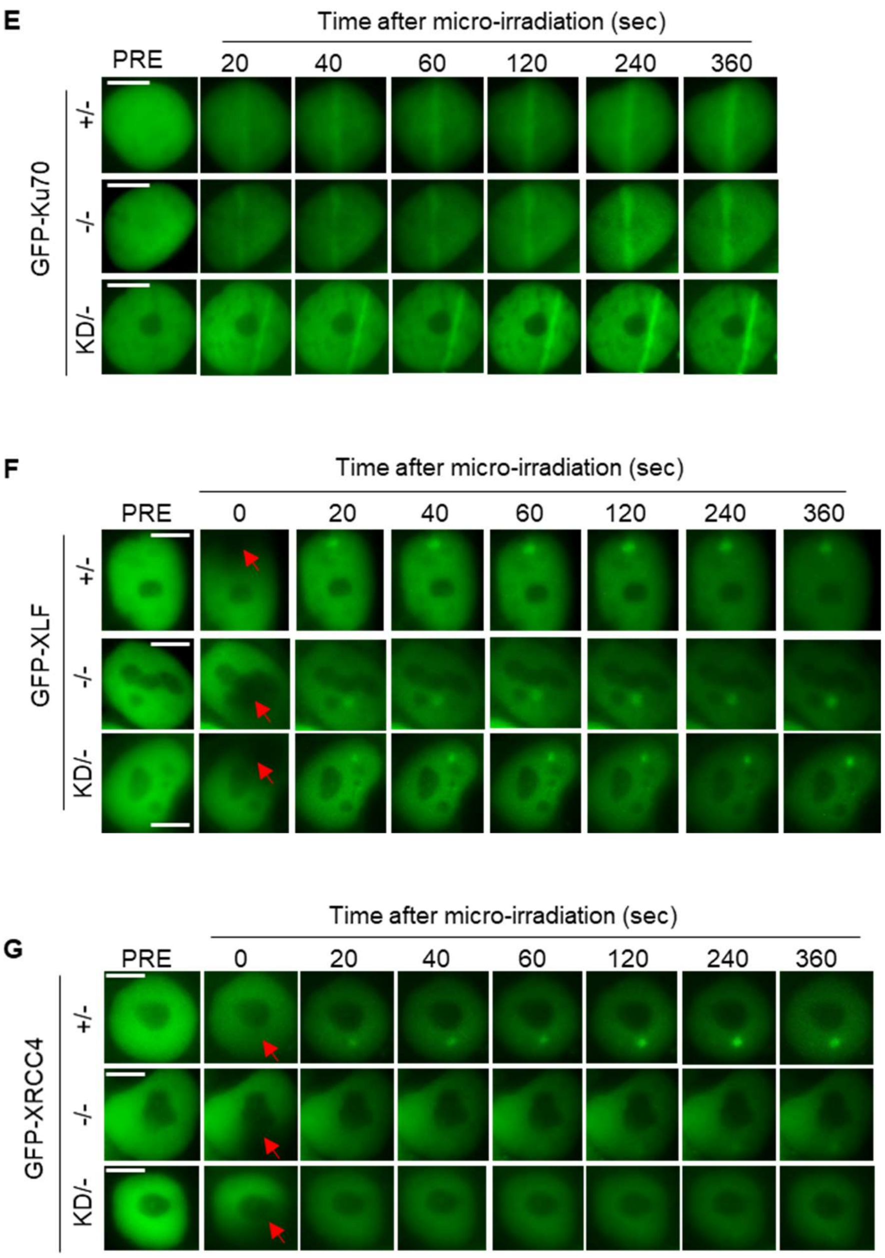

Supplemental Fig. 6 The kinase activity of DNA-PK$_{cs}$ is required to promote recruitment of DDR proteins to DSBs in HCT116 cells. **(A)** Representative images of MDC1 foci in the HCT116 DNA-PK$_{cs}$ +/-, -/- and KD/- cells after 1 Gy irradiation. **(B)** γH2AX foci in HCT116 DNA-PK$_{cs}$ +/+, +/-, -/- and KD/- cells 10 minutes and 30 minutes after a 2-Gy-γ radiation. **(C-G)** representative images of recruitment of GFP-tagged NBS1 **(C)**, EXO1 **(D)**, Ku70 **(E)**, XLF **(F)** and XRCC4 **(G)** to laser-induced DSBs the HCT116 DNA-PK$_{cs}$ +/-, -/-, and KD/- cells. Bar indicates 5 μm.

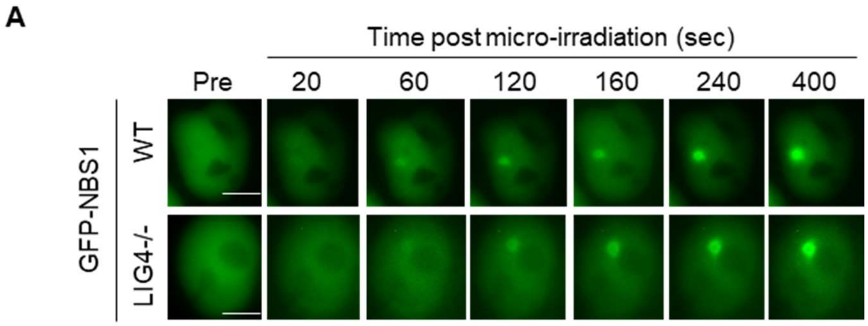

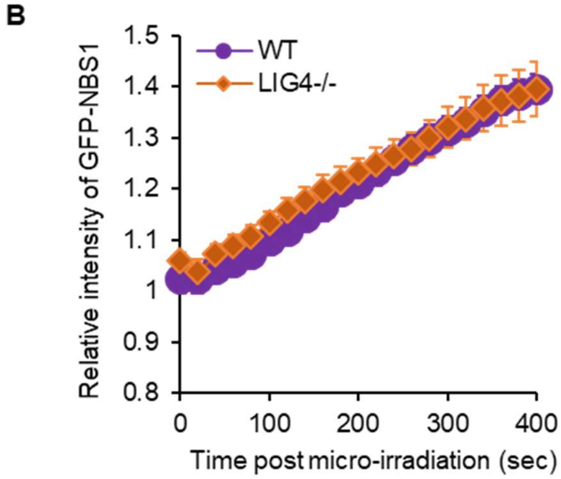

Supplemental Fig. 7 Disruption of NHEJ via loss of DNA Ligase 4 (LIG4 -/-) does not alter GFP-NBS1 recruitment to laser-induced DSBs in HCT116 cells. GFP-NBS1 were transfected to HCT116 wild type and LIG4 null cells, and dynamic accumulation of GFP-NBS1 were monitored and quantified. **(A)** Representative images of GFP-NBS1 recruitment. Bar, 5 µm. **(B)** Relative intensity of GFP-NBS1, showed as Mean ± SEM, were calculated from 20 WT cells and 10 $LIG4^{-/-}$ cells.

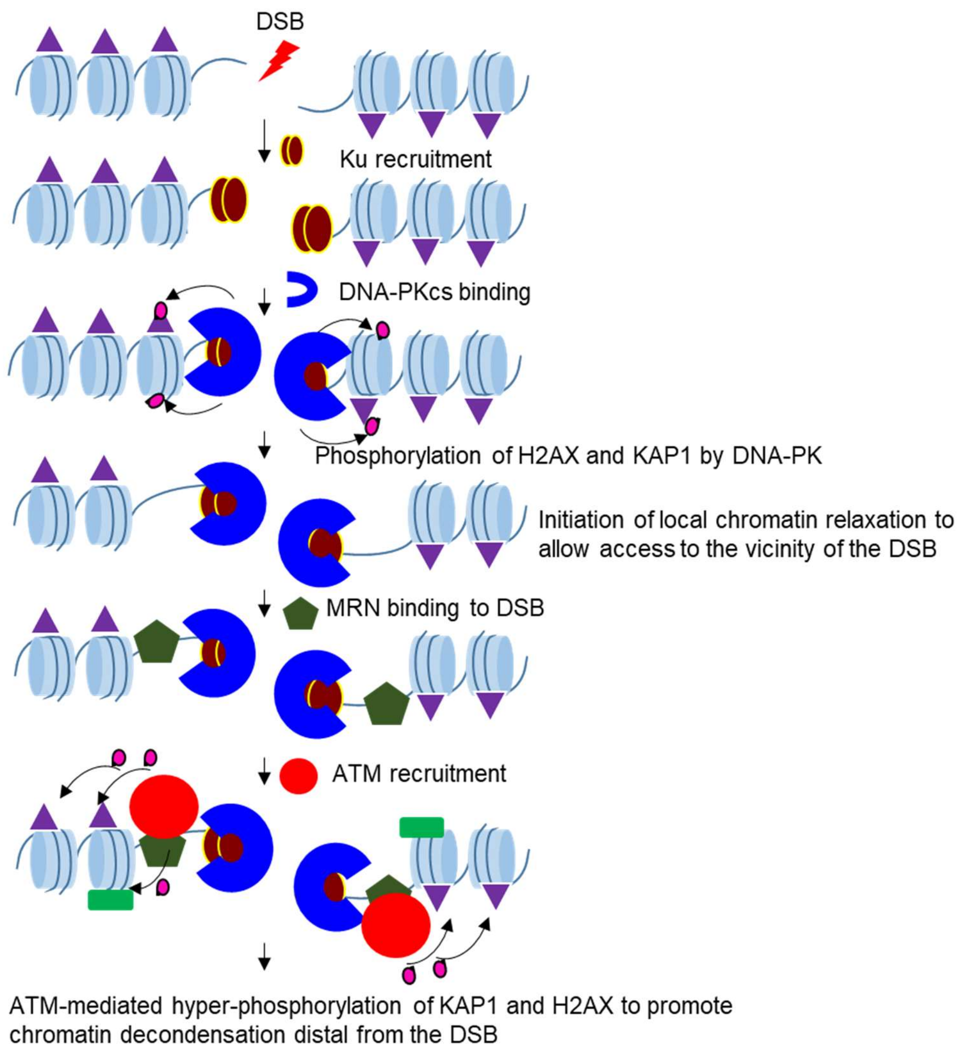

Supplemental Fig. 8 The model for the role of DNA-PK in initial DNA damage response after DSB formation. The Ku70/80 heterodimer recognizes and binds to the DNA lesion immediately after DSB formation, and it further recruits DNA-PK_{cs} for forming DNA-PK complex. DNA-PK phosphorylates H2AX and KAP1 proximal to the DSB site to stimulate local chromatin relaxation, which facilitates rapid recruitment of the DDR machinery at and/or near the DSB site.
